# Validation of the inadequate delivery of oxygen index in an adult cardiovascular intensive care unit

**DOI:** 10.1016/j.xjon.2024.09.006

**Published:** 2024-09-12

**Authors:** Heather Holman, Dimitar Baronov, Jeff McMurray, Arman Kilic, Marc Katz, Sanford Zeigler

**Affiliations:** a Division of Cardiothoracic Surgery, Department of Surgery, Medical University of South Carolina, Charleston, SC; b Etiometry, Inc, Boston, Mass; c Division of Critical Care, Department of Anesthesia, Medical University of South Carolina, Charleston, SC; d Harvey and Marcia Schiller Surgical Innovation Center, Medical University of South Carolina, Charleston, SC

**Keywords:** machine learning, pulmonary artery catheterization, artificial intelligence, hemodynamic monitoring, critical care

## Abstract

**Objective:**

Machine learning (ML) may allow for improved discernment of hemodynamics and oxygen delivery compared to standard invasive monitoring. We hypothesized that an ML algorithm could predict impaired delivery of oxygen (IDO_2_) with comparable discrimination to invasive mixed venous oxygen saturation (SvO_2_) measurement.

**Methods:**

A total of 230 patients not on mechanical circulatory support (MCS) managed with a pulmonary artery catheter (PAC) were identified from 1012 patients admitted to a single cardiovascular intensive care unit (CVICU) between April 2021 and January 2022. Physiologic data were collected prospectively by the data analytics engine. Inadequate delivery of oxygen (IDO_2_) was defined as SvO_2_ ≤50%. Fifty-four patients were used to train the model, which was then validated in 176 patients. Three simulated monitoring situations were constructed by downsampling the physiologic data set to exclude all SvO_2_ sources (scenario A); all PAC data but allowing for SvO_2_ values (scenario B); and all PAC data, including SvO_2_ and cardiac index (CI) (scenario C). The ML platform then calculated the likelihood of IDO_2_ for rolling 30-minute intervals and compared these values against the gold standard SvO_2_ values using receiver operating characteristic (ROC) curve analysis to establish discriminatory power.

**Results:**

A total of 1047 laboratory-validated SvO_2_ values were collected for the validation group. The area under the ROC curve for the IDO_2_ index was 0.89 (95% confidence interval, 0.87-0.91) with the full data set. When blinded to all PAC and SvO_2_ sources, the AUC was 0.78 (95% confidence interval, 0.75-0.81).

**Conclusions:**

The IDO_2_ index is capable of detecting SvO_2_ ≤50% with good discriminatory function in non-MCS CVICU patients in a variety of monitoring situations. Further investigation of IDO_2_ detection and clinical endpoints is needed.

## Graphical abstract

**Figure:**
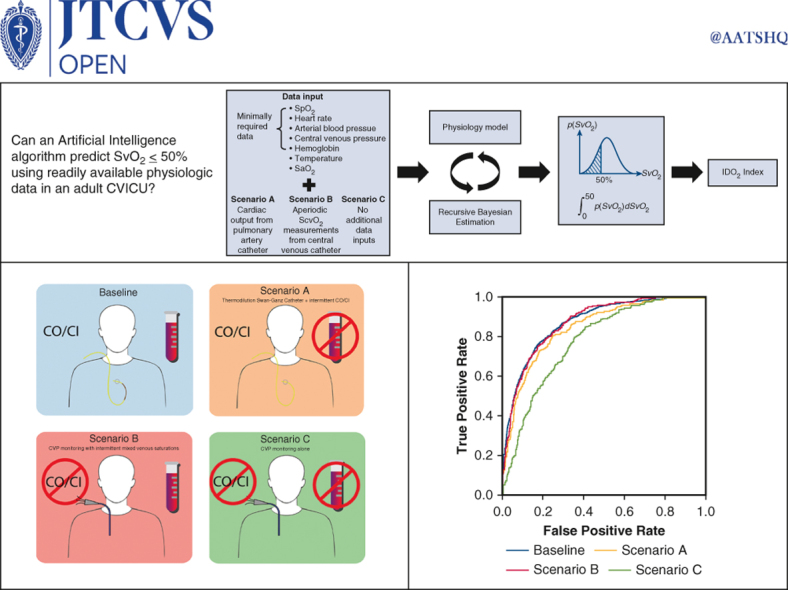

**Figure:**
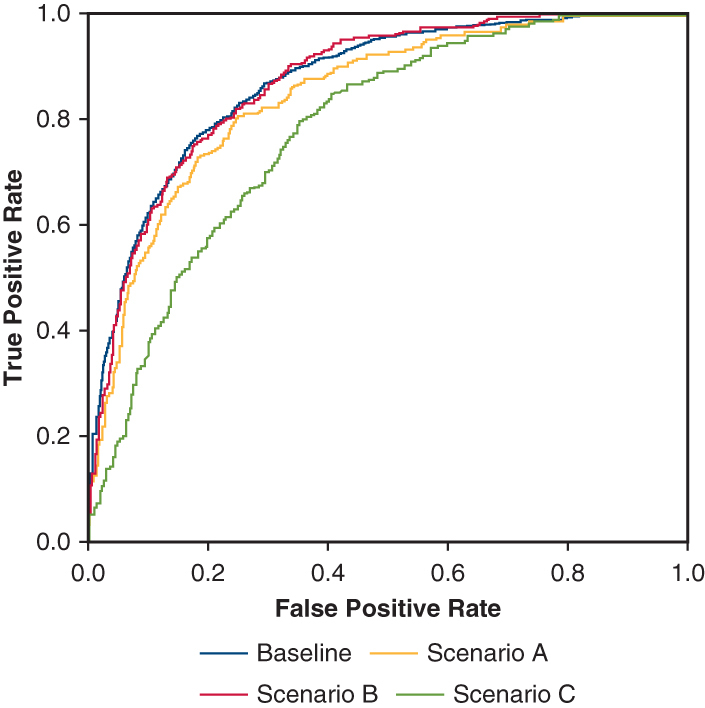
ROC curves of the IDO_2_ index display a high degree of accuracy in predicting SvO_2_ ≤50%.

**Central Message**

The IDO_2_ index is a novel machine learning algorithm that can accurately predict SvO_2_ ≤50% in non-MCS CVICU patients in the absence of both pulmonary artery catheter and ScvO_2_ data.

**Perspective**

The IDO_2_ index machine learning algorithm accurately predicts SvO_2_ ≤50% without the need for continuous oximetry, continuous cardiac output monitoring, or a pulmonary artery catheter. Use of this tool may permit early detection of patient decompensation, allowing for alteration in clinical management and intervention to prevent further deterioration and irreversible end-organ damage.

Predictive analytics and machine learning (ML) have been applied in the healthcare setting to aid complex decision making, risk stratification of patients, and disease diagnosis and prognosis.^1^, ^2^, ^3^ A unique advantage of these modeling systems is that they can synthesize many different types of data over time, generating an objective output. This can reduce human error by alleviating information and interpretation overload and mitigating common biases in decision making (eg, recency bias).^4^ Predictive analytics and ML have yet to be widely adopted in the clinical setting, however.^5^

Previous investigators developed a novel computational physiology algorithm to predict the probability of inadequate delivery of oxygen (IDO_2_) in pediatric patients, by ingesting and processing continuously measured physiologic data and laboratory tests in real time.^6^ The pediatric IDO_2_ index has been calibrated to predict mixed venous oxygen saturation (SvO_2_) <30%, <40%, and <50% to aid the management of a variety of cyanotic and noncyanotic congenital cardiac conditions. Poor oxygen delivery is associated with a poor prognosis in cardiopulmonary disease and shock states and may be associated with adverse outcomes following cardiac surgery.^7^, ^8^, ^9^, ^10^

A similar IDO_2_ index has not been developed or validated in an adult setting. The objective of this study was to develop and validate a computational algorithm that can predict SvO_2_ ≤50% with comparable discrimination to invasive SvO_2_ measurements in the adult cardiovascular intensive care unit (CVICU).

## Patients and Methods

### IDO_2_ Index Algorithm for Detecting SvO_2_ Level

**Figure 1:**
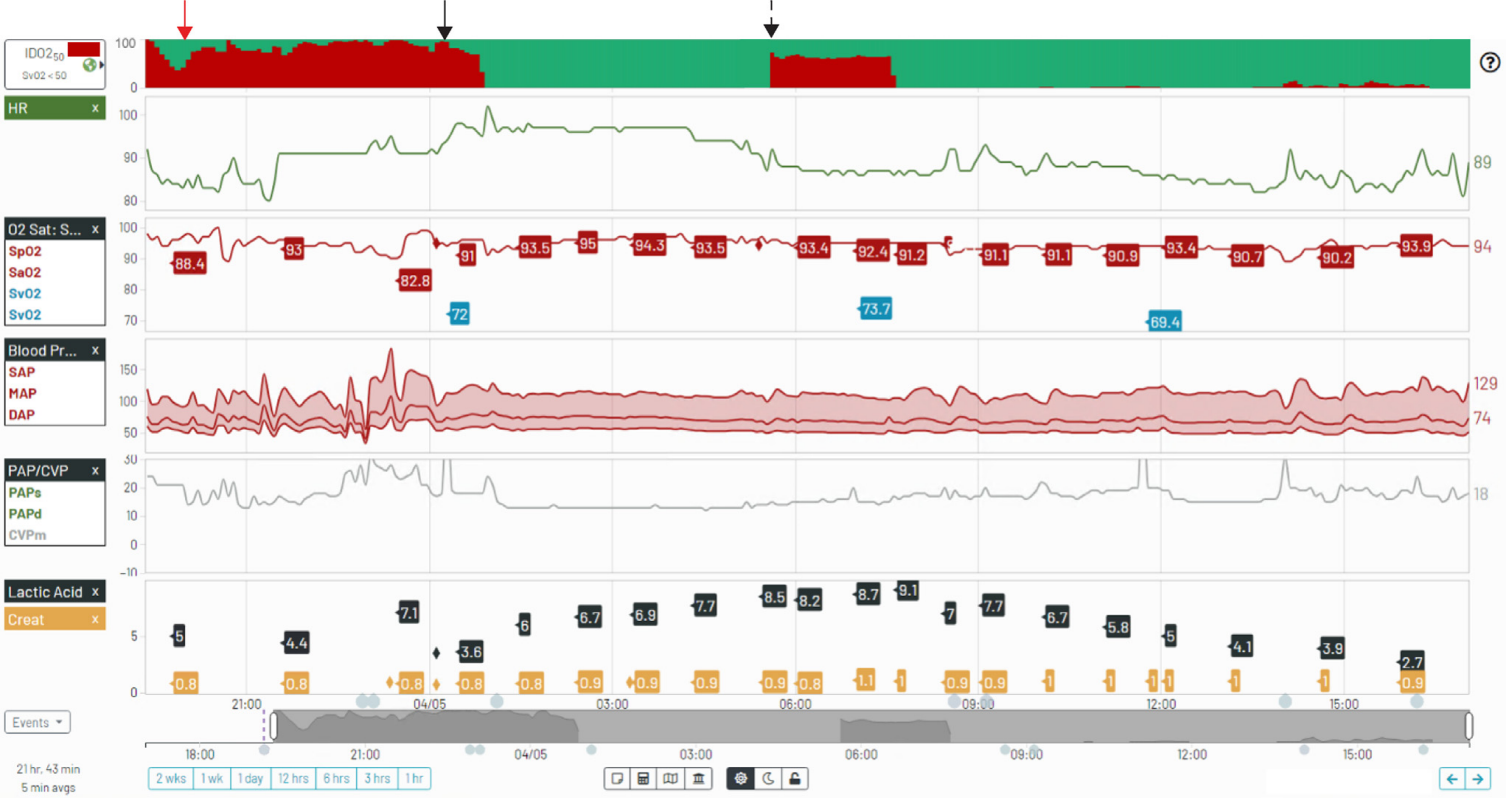
Example case using the impaired delivery of oxygen (*IDO*_*2*_) index. This is a representative image of the Etiometry platform software for an individual patient over 24 hours. This patient underwent a routine 4-vessel coronary artery bypass graft and developed hemodynamic instability following transfer to the cardiovascular intensive care unit. The initial rise in IDO_2_ (*red arrow*) correlated with lactic acidosis. Transesophageal echocardiography demonstrated right ventricular dysfunction, and epinephrine was administered (*black arrow*). The IDO_2_ index and hemodynamics improved immediately. Shortly thereafter, the patient’s lactate level began to rise, but the IDO_2_ index remained low, suggesting a type 2 lactic acidosis related to the epinephrine infusion. Nine hours later, the IDO_2_ index began to rise (*dashed arrow*) as the heart rate decreased. Central venous oxygen saturation (*ScvO*_*2*_) was measured, recalibrating the IDO_2_ algorithm. The low IDO_2_ index was reassuring, precluding the insertion of a pulmonary artery catheter.

**Figure 2:**
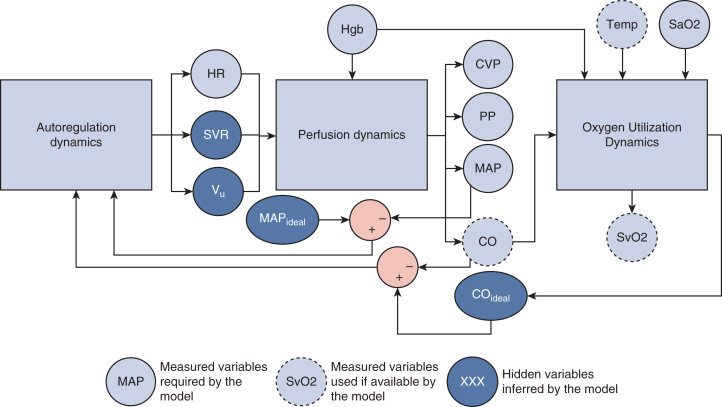
Graphical representation of the impaired delivery of oxygen (*IDO*_*2*_) index hemodynamic model. The model ingests readily obtainable hemodynamic and lab parameters (*unshaded circles*) and infers other values (*shaded circles*) based on mathematical descriptions of the various physiologic relationships (*arrows*) and local dynamics (*boxes*). Note that mixed venous oxygen saturation (*SvO*_*2*_) refers to continuous oximetry numbers in real time or the most recent laboratory confirmed SvO_2._

The IDO_2_ index is a multivariate algorithm designed to analyze, in near-real time, monitored patient parameters, demographic data, and laboratory results to continuously quantify the probability that a patient’s SvO_2_ is below a specific critical value^6^ (Figure 1). The algorithm uses a static computational physiology model containing more than 20 ordinal differential equations describing the factors that influence human oxygen delivery (Figure 2), along with computational feedback loops to account for patterns in individual physiology, correction of measurement error, and random variation. A recursive Bayesian estimation is applied to the physiology model to interpret the acquired data in a continuous assessment of the patient state.^11^ A full description of the IDO_2_ computational algorithm has been published previously.^6^

**Figure 3:**
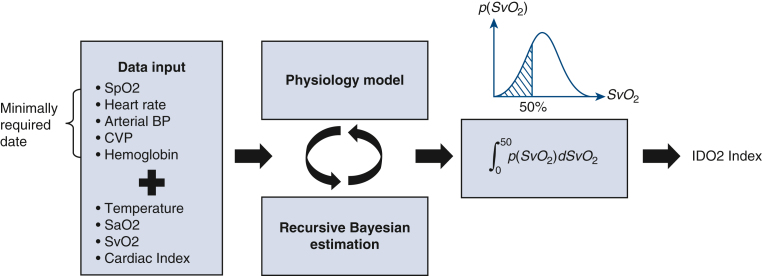
Simplified description for calculation of the impaired delivery of oxygen (*IDO*_*2*_) index. The IDO_2_ index was calculated from a multivariate algorithm that used a physiologic model generated from specific data inputs, such as pulse oximetry, heart rate from either electrocardiography or pulse oximetry, invasive arterial blood pressure, central venous pressure, and hemoglobin. Additional physiologic data can be added to the model to improve its accuracy. This physiologic model underwent recursive Bayesian estimation to interpret the acquired data into a continuous assessment of the patient’s state.

By ingesting physiologic data as input variables, the model produces a probability distribution function for SvO_2_ accounting for patient-to-patient variation and measurement uncertainty. For this study, the model was required to have the input of a minimal data set and also incorporates additional commonly measured hemodynamic data when present (Figure 3). The model then calculates a probability density plot of the predicted SvO_2_ value. We defined the IDO_2_ index as the cumulative probability of SvO_2_ being ≤50%. Thus, increasing IDO_2_ index values would indicate that the true SvO_2_ was ≤50% with greater certainty.

If the minimum data set is not acquired, no IDO_2_ index computation is returned. The minimum necessary data streams are:

• Pulse oximetry every 5 seconds
• Heart rate from either electrocardiography or pulse oximetry every 5 seconds
• Invasive arterial blood pressure every 5 seconds
• Central venous pressure every 5 seconds
• Hemoglobin sampled at least once every 24 hours.

The algorithm incorporates additional data, when present, to refine the probability density calculation. These additional data include:

• Temperature
• Continuous and/or aperiodic cardiac index (CI)
• SvO_2_ sourced from either laboratory results or a continuous oximetry catheter.

In this study, only patients with recordings of all the foregoing data were included.

### Data Collection

This study was approved by the Medical University of South Carolina Institutional Review Board (Pro00112195, approved August 21, 2021), which waived the requirement for informed consent. Deidentified data for all patients admitted to the Medical University of South Carolina’s CVICU between January 28, 2021, and January 31, 2022, with a pulmonary artery catheter (PAC) and any mixed venous co-oximetry laboratory results were included in the study, independent of age, race, or sex. Data were collected only from patients who satisfied the full data set (Figure 3). PAC placement, model, and duration of placement were at the treating clinician’s discretion, as was SvO_2_ monitoring interval. Data was captured via a software link making HL7 data packets available to the Etiometry T3 analytics engine.

Patients requiring mechanical circulatory support (MCS) during any intensive care unit (ICU) admission were excluded from the analysis by manual chart review. The computational model for the IDO_2_ index relies on an assumption of normal pulsatile hemodynamic physiology, as it relies on pulse pressure–dependent calculations of stroke volume to determine blood flow and cannot incorporate external influences on blood flow. As such, using the IDO_2_ index is contraindicated for patients receiving MCS.

The resultant data were then divided into 2 temporally independent data sets. The first set was collected from January 28, 2021, to April 9, 2021 (n = 54 patients; 404 SvO_2_ reference values) and used for algorithm development. The second data set, collected from April 10, 2021, to January 31, 2022 (n = 176 patients; 276 reference values), was used to validate the algorithm performance. Only the results from the second, independent data set from April 10, 2021, to January 31, 2022, are reported in this study.

### Analysis Methodology

The IDO_2_ index was computed retrospectively on the deidentified patient data. Continuous data streams (ie, transduced pressures and continuous oximetry) were sampled every 5 seconds to create discrete values. For each patient, all mixed venous blood gases and their associated time stamps were identified, and the average values of the index were computed for 30 minutes leading to, but excluding, the time stamp of the gold standard (SvO_2_). For the purpose of creating receiver operating characteristic (ROC) curves for the IDO_2_ index, the gold standard value (SvO_2_) was dichotomized into true- positive and true-negative test results: positive for inadequate oxygen delivery corresponding to SvO_2_ ≤50% and negative for inadequate oxygen delivery corresponding to SvO_2_ >50%.

The IDO_2_ index paired with the respective SvO_2_ laboratory values was used to conduct the following performance analysis:

• Evaluate the IDO_2_ index’s discriminatory power by the area under the ROC curve (AUC) for detecting SvO_2_ <50% by the IDO_2_ index.
• Evaluate the IDO_2_ index performance robustness under different monitoring scenarios by downsampling the original data set and assessing the AUC for each scenario, each simulating different potential monitoring scenarios:

Scenario A: blinded to all SvO_2_ data either from laboratory results or continuous oximetry.

Scenario B: blinded to PAC data, such as cardiac output and continuous oximetry, while still ingesting supplemental aperiodic data from SVO_2_ measurement.

Scenario C: blinded to all PAC data and all sources of venous oxygen saturation.

KJL Scenario A simulates monitoring a patient with a bolus thermodilution PAC that lacks continuous oximetry, between CO measurements and laboratory-derived SvO2 measurements. Scenario B simulates a patient without a PAC, but with intermittent central venous oxygen saturation (ScvO_2_) sampling via a central venous catheter. Finally, scenario C represents a patient with a central venous catheter, but no PAC or any ScvO_2_ measurements.

The ROC curves for each scenario were generated by sweeping over all possible IDO_2_ index values and computing the true positive and false positive rate at each value. The mean AUC for each curve was computed by integrating the curve using trapezoidal integration, while the 95% confidence intervals were calculated by using bootstrapped AUC samples derived from random subsets selected with replacement from the original data set.

## Results

### Patient Demographics

**Figure 4:**
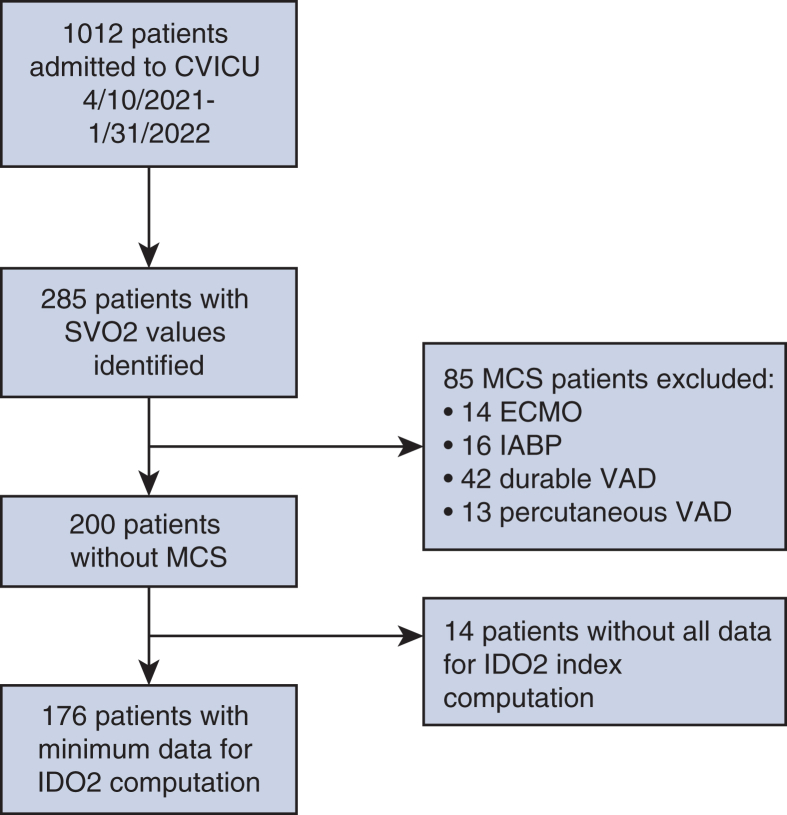
Study design and inclusion flowchart.

A total of 1012 patients were admitted in the CVICU between April 10, 2021, and January 31, 2022, of whom 285 patients had SvO_2_ laboratory values. Of these, 85 were excluded due to the presence of MCS and 14 were excluded due to a lack of the minimum data required for index computation. Figure 4 summarizes the specific exclusion numbers for each category. Receipt of 4 types of MCS excluded patients: extracorporeal membrane oxygenation, intra-aortic balloon pump, durable ventricular assist device, and Impella. Table 1 summarizes the demographic data of the 176 patients included in the study.

**Table 1:** Patient demographics and descriptive statistics

Characteristic	Value
Male sex, n (%)	121 (69)
Postoperative status, n (%)	137 (78)
Age, y, median (range)	64.6 (54.4-73.3)
CI measured, n (%)	119 (68)
Continuous CI, n (%)	79 (45)
Aperiodic CI, n (%)	40 (23)
Continuous oximetry catheter, n (%)	84 (48)
SvO_2_ labs evaluated per patient, median (range)	3 (1-5)

*CI*, Cardiac index; *SvO*_*2*_, mixed venous oxygen saturation.

### IDO_2_ Index Performance

**Figure 5:**
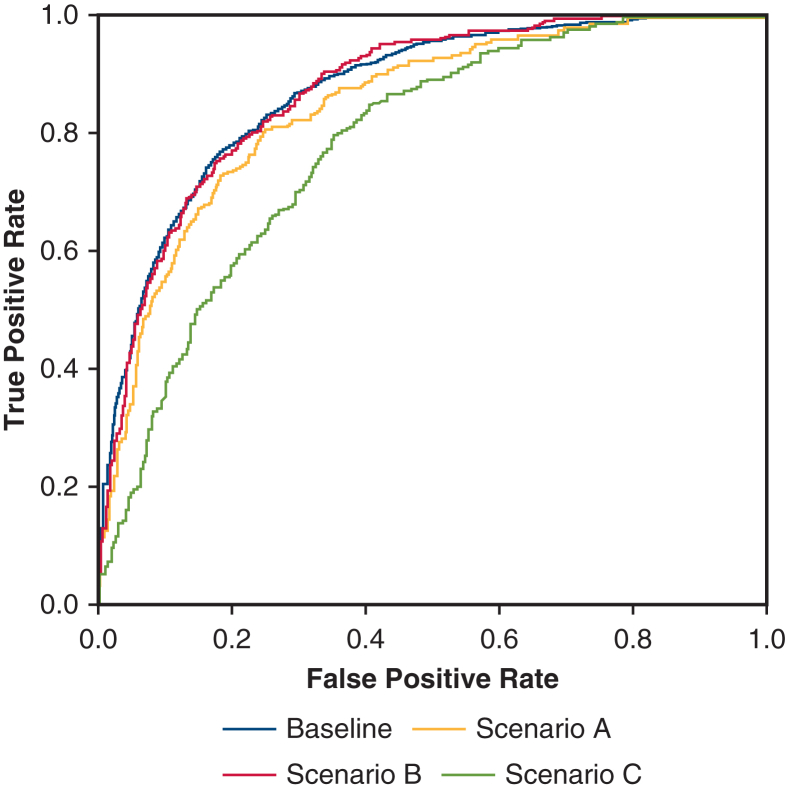
Receiver operator characteristic (*ROC*) curves for the impaired delivery of oxygen (*IDO*_*2*_) index. ROC curves were generated for the baseline (presence of mixed venous oxygen saturation [*SvO*_*2*_] and cardiac index [*CI*] monitoring) and the downsampling scenarios A, B, and C. Scenario A: no SvO_2_ monitoring; scenario B: no CI monitoring; scenario C: no CI or SvO_2_ monitoring. Scenarios A and B demonstrated similar performance as the baseline scenario. Importantly, scenario C still maintained a high degree of accuracy in detecting SvO_2_ ≤50%.

The ROC in scenarios A and B, which included either CI or SvO_2_ monitoring, closely tracked the baseline scenario. A decline in model performance observed in scenario C, which lacked ScvO_2_ and CI data (Figure 5). Baseline and individual scenario AUC values are summarized in Table 2. The AUC was 0.89 (95% confidence interval, 0.87-0.91) for the baseline scenario, 0.84 (95% confidence interval, 0.82-0.87) for scenario A, 0.87 (95% confidence interval, 0.84-0.89) for scenario B, and 0.78 (95% confidence interval, 0.75-0.81) for scenario C.

**Table 2:** ROC curve calculations and SvO_2_ laboratory values

Scenario	AUC (95% CI)	SvO_2_ <50%, n (total SvO_2_ laboratory values, n)
Baseline	0.89 (0.87-0.91)	257 (1047)
Scenario A	0.84 (0.82-0.87)	256 (1042)
Scenario B	0.87 (0.84-0.89)	256 (1042)
Scenario C	0.78 (0.75–0.81)	256 (1045)

Calculations of the area under the ROC curve and 95% confidence interval of the ROC curves were generated for the baseline and downsampling scenarios. Baseline: presence of SvO_2_ and cardiac index (CI) monitoring; scenario A: no SvO_2_ monitoring; scenario B: no CI monitoring; scenario C: no CI or SvO_2_ monitoring. *ROC*, Receiver operating characteristic; *SvO*_*2*_, mixed venous oxygen saturation.

## Discussion

The IDO_2_ index is capable of detecting SvO_2_ ≤50% with good discrimination in a mixed medical and surgical CVICU, excluding patients on MCS. The data collected for this study were used to gain Food and Drug Administration approval for the IDO_2_ index in adult ICU populations. While clinicians should be able to identify obvious hypotension, low cardiac output, and shock states, subtle variations in lab values, hemodynamics, and peripheral oxygen saturation may signal impaired oxygen delivery before the onset of shock and between periodic measurements of blood oxygen content and cardiac output. The IDO_2_ index model can monitor for impaired oxygen delivery continuously in various ICU monitoring settings at 5-second intervals.

In prior studies, an elevated IDO_2_ index predicted cardiac arrest 120 minutes prior to the arrest in neonates after cardiopulmonary bypass surgery, and predicted failure to wean vasoactive medications in postoperative pediatric cardiac surgery patients.^4^^,^^12^ In a noncardiac pediatric sepsis population, a high IDO_2_ up to 12 hours after hospital admission was associated with major adverse events and worse outcomes.^13^ Additionally, a low IDO_2_ index may rule out a risk for cardiac arrest in critically ill pediatric patients who are classified as high-risk for cardiac arrest by standard clinical evaluation.^14^ Thus, these studies demonstrate the utility of measuring IDO_2_ in critically ill patients and its potential for altering clinical management. Our study did not attempt to correlate clinical outcomes with the IDO_2_ index in adults.

Importantly, our model maintains its predictive capability even when PAC data or venous saturation data are removed. The use of PACs varies widely across institutions, and there has been a decline in their use over the last several decades due to controversy over their clinical benefit.^15^^,^^16^ Some ICU studies have shown no difference in mortality benefit or hospital length of stay with the use of PACs,^15^^,^^16^ although they are still used in many cardiac-oriented ICUs. Although reported rates of complications with PACs are <4%, there is still a possibility of atrial and ventricular arrythmias during insertion and removal, as well as the potential for infections, pulmonary artery rupture, pulmonary infarction, and venous thrombosis.^15^ Moreover, owing to global supply chain issues stemming from the COVID-19 pandemic and its aftermath, many ICUs intermittently lost access to their usual type of PAC, precluding continuous oximetry or cardiac output monitoring.

Scenario A represents a patient with a thermodilution PAC that is incapable of measuring continuous SvO_2_. Scenario B represents a patient with a central venous catheter and intermittent ScvO_2_ monitoring. Scenario C represents a patient with a central line used to measure central venous pressure only. In critically ill patients requiring an invasive PAC, the IDO_2_ index could provide continuous hemodynamic data for heightened surveillance when continuous oximetry is not available or when a PAC is contraindicated (scenarios A and B). On the other hand, in patients at lower operative risk or patients who are recovering hemodynamically, the IDO_2_ index could provide a more robust evaluation of oxygen delivery to accelerate weaning of hemodynamic agents via a central venous catheter only (scenario C). Incorporation of this model at bedside may allow for more accurate tracking of oxygen delivery regardless of the presence or style of PAC.

ScvO_2_ laboratory measurements are subject to inaccuracies and are an imperfect surrogate for SvO_2_ measurements obtained from PACs. They vary widely based on patient position, location of sample draw, blood oxygen consumption, and distribution of blood flow.^17^^,^^18^ ScvO_2_ measurements are not necessary to maintain the accuracy of the IDO_2_ index with this algorithm (scenario A or C). However, if a patient has frequent ScvO_2_ measurements due to a clinical concern, these data can be added to the model and improve the accuracy of the IDO_2_ index and the predictability of patient decompensation (scenario B).

Previous studies in pediatric ICUs have demonstrated that accumulated time with high IDO_2_ can predict cardiac arrest, failure to wean off vasoactive medications, and adverse events in critically ill children.^4^^,^^12^^,^^13^ While this study demonstrates that the IDO_2_ index accurately predicts SvO_2_ ≤50 in an adult CVICU, future studies will need to demonstrate whether the use of this model can prevent patient decompensation and end-organ failure or accelerate liberation from vasoactive support. Advanced knowledge of depressed SvO_2_ prior to the onset of hypotension, desaturation, or heart rate disturbances may allow physicians to intervene sooner, with a goal of recognizing and stopping decompensation before end organ-damage can occur.

Although ML algorithms have yet to be widely used in daily clinical practice, other models have demonstrated their benefit in an ICU setting. For example, the model developed by Rojas and colleagues was found to more accurately predict ICU readmission compared to the current predictive tools (Stability and Workload Index for Transfer and the Modified early Warning Score).^19^^,^^20^ Additionally, the ML model developed by Ryan and colleagues^21^ predicted acute kidney injury approximately 13 hours prior to clinical detection. Furthermore, Choi and colleagues^22^ compared ML algorithms to conventional scoring systems and demonstrated that ML outperformed the conventional scoring systems in predicting in-hospital mortality in adult ICU patients.

### Limitations

This study is limited by its use of a CVICU population, and the results might not be reproducible in other critical care, stepdown, or general care settings. Patients requiring MCS at any point in their hospitalization were excluded from the study, and these patients may represent an important clinical entity with more severe perturbations in SvO_2_. Because only patients admitted with a PAC were studied, and the decision to place a PAC was made at the discretion of the attending physician, many lower-risk patients were excluded. Further studies should examine whether use of the IDO_2_ index via bedside display leads to improved patient outcomes over standard hemodynamic, laboratory, and clinical examination and should validate the findings in other inpatient care settings.

The scenario B simulation is limited by a lack of ScvO_2_ measurements. Laboratory-measured SvO_2_ was used as a surrogate, and this value is more accurate than ScvO_2_ in calculating oxygen consumption. Our lab currently does not distinguish between ScvO_2_ and SvO_2_ measurements. Confirmation of the model’s efficacy using ScvO_2_ would require simultaneous ScvO_2_ and SvO_2_ measurements, which was not feasible in this study.

The model itself is limited by assumptions regarding normal variations in baseline physiology. For example, arterial stiffness tends to increase over time. Although age is incorporated into the model, there is no direct input to account for arterial stiffness, which adds uncertainty to the model. Further studies are needed to validate the model in specific populations expected to have differences in ventriculoarterial coupling (ie, heart failure, aortic dissection, end-stage renal failure). Another limitation of the model is that it does not report which inputs are most heavily impacting changes in the IDO_2_ index. Thus, if a patient is noted to have an elevated IDO_2_ index, it is up to the person at the bedside to use their clinical judgment determine the root causes of impairment and what physiologic parameters they can alter to improve oxygen delivery.

Although we chose SvO_2_ of 50% as the reference value to calibrate the IDO_2_ index for adults, this might not be the optimal value. We chose this value because the IDO_2_ index has been used and has received Food and Drug Administration approval in pediatric populations at threshold SvO_2_ levels of 30%, 40%, and 50%. The lower thresholds are relevant for children with cyanotic heart disease but are not necessary in an adult CVICU. Since the 50% threshold had been developed and investigated in a pediatric setting, we modified the pediatric IDO_2_ index to build on this prior work. In addition, we wanted to choose a threshold that would reflect a meaningful clinical deterioration and not contribute to alarm fatigue. Finally, the cardiac surgery population includes a proportion of patients with advanced heart failure, whose baseline SvO_2_ is often <60%. The most appropriate threshold value likely depends on an individual patient’s baseline state and comorbid conditions. Setting the threshold value higher likely would provide earlier warning of decompensation, but with more false-positive results. This would lead to a less accurate predictive model and likely contribute to alarm fatigue when using the model. Determining the most useful disease-state specific threshold values is an area for future research.

## Conclusions

**Figure 6:**
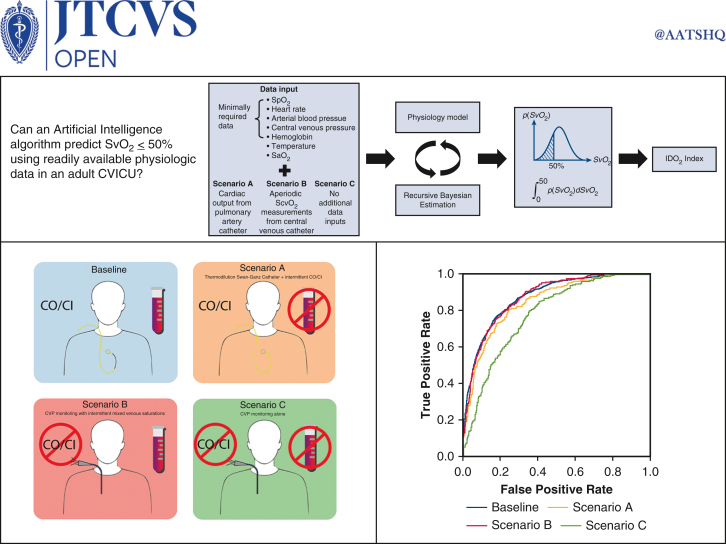
Graphical abstract.

The IDO_2_ index ML algorithm can accurately predict SvO_2_ ≤50% in non-MCS CVICU patients in a variety of monitoring situations (Figure 6). Importantly, the model maintains a high degree of accuracy in the absence of both PAC and ScvO_2_ data. Further study is needed to evaluate clinical utility of such a model in practice.

## Conflict of Interest Statement

Etiometry, Inc funded this research and provided material assistance in the collection of data. D.B. is a Founder and Chief Technology Officer of Etiometry, Inc, and owns shares in the company. D.B. assisted in the development of the IDO_2_ index and the analyses performed in this study. A.K. is a speaker/consultant for Abbott, Abiomed, 3ive, and LivaNova. M.K. is a consultant for Abbott Vascular, Boston Scientific, Clearview Scientific, Edwards Lifesciences, and Medtronic but has no financial relationship with them. S.Z. is an unpaid consultant for Etiometry, Inc, is a consultant for Terumo Aortic and a speaker for Endospan. H.H. and J.M. reported no conflicts of interests.

The *Journal* policy requires editors and reviewers to disclose conflicts of interest and to decline handling or reviewing manuscripts for which they may have a conflict of interest. The editors and reviewers of this article have no conflicts of interest.
